# The *mutL* Gene as a Genome-Wide Taxonomic Marker for High Resolution Discrimination of *Lactiplantibacillus plantarum* and Its Closely Related Taxa

**DOI:** 10.3390/microorganisms9081570

**Published:** 2021-07-23

**Authors:** Chien-Hsun Huang, Chih-Chieh Chen, Yu-Chun Lin, Chia-Hsuan Chen, Ai-Yun Lee, Jong-Shian Liou, Chun-Tao Gu, Lina Huang

**Affiliations:** 1 Bioresource Collection and Research Center, Food Industry Research and Development Institute, 331 Shih-Pin Rd, Hsinchu 30062, Taiwan; ayl@firdi.org.tw (A.-Y.L.); ljs@firdi.org.tw (J.-S.L.); hln@firdi.org.tw (L.H.); 2 Institute of Medical Science and Technology, National Sun Yat-sen University, Kaohsiung 80424, Taiwan; chieh@imst.nsysu.edu.tw; 3 Rapid Screening Research Center for Toxicology and Biomedicine, National Sun Yat-sen University, Kaohsiung 80424, Taiwan; 4 Livestock Research Institute, Council of Agriculture, Executive Yuan, Tainan 71246, Taiwan; hiujj@tlri.gov.tw (Y.-C.L.); joyce729@mail.tlri.gov.tw (C.-H.C.); 5 College of Life Sciences, Northeast Agricultural University, Harbin 150030, China; ctgu1977@hotmail.com

**Keywords:** comparative genome sequence analysis, genome-wide target, species-specific identification, *Lactobacillus plantarum* group, *Lactiplantibacillus*

## Abstract

The current taxonomy of the *Lactiplantibacillus plantarum* group comprises of 17 closely related species that are indistinguishable from each other by using commonly used 16S rRNA gene sequencing. In this study, a whole-genome-based analysis was carried out for exploring the highly distinguished target genes whose interspecific sequence identity is significantly less than those of 16S rRNA or conventional housekeeping genes. In silico analyses of 774 core genes by the cano-wgMLST_BacCompare analytics platform indicated that *csbB*, *morA*, *murI*, *mutL*, *ntpJ*, *rutB*, *trmK*, *ydaF*, and *yhhX* genes were the most promising candidates. Subsequently, the *mutL* gene was selected, and the discrimination power was further evaluated using Sanger sequencing. Among the type strains, *mutL* exhibited a clearly superior sequence identity (61.6–85.6%; average: 66.6%) to the 16S rRNA gene (96.7–100%; average: 98.4%) and the conventional phylogenetic marker genes (e.g., *dnaJ*, *dnaK*, *pheS*, *recA*, and *rpoA*), respectively, which could be used to separat tested strains into various species clusters. Consequently, species-specific primers were developed for fast and accurate identification of *L. pentosus, L. argentoratensis*, *L. plantarum*, and *L. paraplantarum*. During this study, one strain (BCRC 06B0048, *L. pentosus*) exhibited not only relatively low *mutL* sequence identities (97.0%) but also a low digital DNA–DNA hybridization value (78.1%) with the type strain DSM 20314^T^, signifying that it exhibits potential for reclassification as a novel subspecies. Our data demonstrate that *mutL* can be a genome-wide target for identifying and classifying the *L. plantarum* group species and for differentiating novel taxa from known species.

## 1. Introduction

Following reclassification of *Lactobacillus* according to physiological criteria, clade-specific signature genes, core genome phylogeny, organism ecology, and pairwise average amino acid identity, this varied genus now comprises of 25 genera, with 23 of them being novel [1]. This change to the taxonomy has affected major probiotic *Lactobacillus* species; for example, *Lactobacillus plantarum* has become *Lactiplantibacillus plantarum*. Because the new genera suggested for this group all still begin with an “L”, the abbreviated genus and species names are unchanged. *L. plantarum* is a versatile lactic acid bacteria (LAB) species that one can isolate from different fermented foods and the human gastrointestinal tract [2,3]. Certain *L. plantarum* strains, some of which are recognized probiotics, are generally recognized as safe and have a qualified presumption of safety status [4,5], designations that are widely applied in food, agriculture, and industrial fermentation [6].

Relevant phylogenomic analyses have classified *L. plantarum* as a member of the *L. plantarum* group along with the following 16 species: *L.* *argentoratensis*, *L. daoliensis*, *L. daowaiensis*, *L. dongliensis*, *L. fabifermentans*, *L. garii*, *L. herbarum*, *L. modestisalitolerans*, *L. mudanjiangensis*, *L. nangangensis*, *L.* *paraplantarum*, *L.* *pentosus*, *L. pingfangensis*, *L. plajomi*, *L. songbeiensis*, and *L. xiangfangensis* [1,7,8]. Among these species, *L. argentoratensis*, *L. paraplantarum*, and *L. pentosus* are the most closely related to *L. plantarum*; they not only share extremely high sequence identities with full-length 16S rRNA genes (100%, 99.7%, and 99.9%, respectively) but also numerous common phenotypic characteristics [9]. Consequently, substantially conserved protein–coding housekeeping genes with more favorable discrimination power have been used to resolve the relationships among closely related bacterial species groups [10]. Researchers have mostly relied on sequence analysis of protein-coding housekeeping genes such as RNA polymerase alpha subunit (*rpoA*) and phenylalanyl t-RNA synthase alpha subunit (*pheS*) to differentiate LAB species, which are closely related, of the *Lactobacillus*, *Leuconostoc*, *Weissella*, *Enterococcus,* and *Pediococcus* genera [11,12,13,14,15,16,17,18,19,20,21]. A previously executed piece of research recommended the use of no less than two additional phylogenetic markers for accurate isolate identification, particularly for the description of novel species in the *Lactobacillus* genus [22]. In addition, higher-level taxonomy and phylogeny can be determined through the execution of multilocus sequence analysis (MLSA) involving the concatenation of several housekeeping genes (typically five or more; [23]). Nevertheless, MLSA is known to exhibit a few disadvantages, including the absence of a universal threshold for species definition, the lack of a common set of genes, and the excessive expense, labor, and time necessary when using various series of housekeeping genes [24,25,26,27,28]. In the current genomic era, prokaryotic taxonomy depends mainly on comparative genome sequence analysis [29,30,31,32]. As a minimum standard requirement, researchers have proposed species-level delineation through the calculation of overall genome-related indices, including average nucleotide identity (ANI) and digital DNA–DNA hybridization (dDDH; [33,34,35]). Although whole-genome sequencing (WGS) constitutes a credible taxonomic information source, it can still be costly, time-consuming, and difficult to apply. Thus, developing a rapid and cost-effective identification and discrimination method that mainly relies on the most informative region of genome sequence data is reasonable and necessary.

In this study, we explored genome-wide taxonomic markers with high discrimination power for accurately identifying species in the *L. plantarum* group. We executed the direct sequencing of polymerase chain reaction (PCR) products through Sanger’s method for resolution validation by targeting the most distinguished phylogenetic marker of *mutL*. Moreover, a novel *L. pentosus* subspecies was differentiated according to genome-based and phenotypic characteristics.

## 2. Materials and Methods

### 2.1. Genome-Based Mining of Taxonomic Markers for Species-Level Discrimination within L. plantarum Group

The standalone version of the cano-wgMLST tool [36] was applied in this genome-wide analysis. Nine whole genomes belonging to *L.* *argentoratensis*, *L. garii*, *L. herbarum*, *L. modestisalitolerans*, *L. nangangensis*, *L.* *paraplantarum*, *L.* *pentosus*, *L. plajomi*, and *L. xiangfangensis* were annotated using Prokka [37] to identify the coding sequences. The pan genome analysis was performed using Roary [38] with a 75% minimum BLASTP percentage identity for the loci identification in species-level. BLAST [39] was then used for allele calling with a minimum identity of 90% and coverage greater than 90% for the locus assignment (presence/absence profile) and exact match for the allele assignment (allele profile). The loci with the largest allele size were selected as the candidate taxonomic markers for species-level discrimination, and their pairwise sequence identity matrices were calculated using Clustal Omega [40].

### 2.2. L. plantarum Group Strains and Culture Conditions

The Bioresource Collection and Research Center (BCRC, Hsinchu, Taiwan) served as the source of most of the reference strains employed in this study (Table 1). The bacterial strains were cultivated on lactobacilli MRS agar (Difco Laboratories, Detroit, MI, USA) anaerobically for 24 h at 37 °C. Genomic DNA was extracted using the DNeasy kit (Qiagen, Valencia, CA, USA) following the manufacturer’s protocols.

### 2.3. Degenerate PCR Primer Design, Nucleotide Sequencing and Phylogenetic Analysis on mutL Gene

On comparison with the *mutL* gene sequence from the whole genome among the species of the *L. plantarum* group, the degenerate primers, LpmutL-F (5′-TSGAYGTSAAYGTKCAYCC-3′) and LpmutL-R (5′-ATGYGGRCARTTRAANGGAT-3′), were designed and targeted to the conserved region. PCRs were performed using 23 μL of sterile MilliQ water, 3 μL of 10× PCR buffer, 0.5 μL of denucleoside triphosphates (10 mM), 1.2 μL of forward primer (10 mM), 1.2 μL of reverse primer (10 mM), 1.5 U of *Taq* DNA polymerase (DreamTaq, Thermo Scientific, Waltham, MA, USA), and 1 μL of template DNA (100 ng/μL). The thermal protocol consisted of the following conditions: initial strand denaturation at 94 °C for 5 min, followed by 30 cycles at 94 °C for 1 min, 58 °C for 1 min, and 72 °C for 1 min, with a final extension step at 72 °C for 7 min. The resulting amplicons were purified using a QIA quick PCR Purification Kit (Qiagen, Inc., Valencia, CA, USA) and sequenced using a BigDye Terminator v3.1 cycle-sequencing kit on a 3730 DNA Analyzer (Applied Biosystems and Hitachi, Foster City, CA, USA). All sequences were aligned using ClustalX version 1.8 [41], and the sequence identities were calculated using Clustal Omega. A phylogenetic tree was reconstructed using the software package mega version 7.0 [42]. The number of haplotypes was calculated using DnaSP version.5.1 [43].

### 2.4. Species-Specific Primer Design and Direct PCR-Based Identification

*L. argentoratensis*–, *L. paraplantarum*–, *L. pentosus*–, and *L. plantarum*–specific PCR primer sets were determined on the basis of *mutL* sequences using VectorNTI version 9.0 (Invitrogen, Carlsbad, CA, USA). Species-specific PCR testing was conducted on all *L. plantarum* group strains and 12 nontarget strains (Table 1). Regarding the steps constituting the thermal cycler protocol, initial strand denaturation was executed at 94 °C for 5 min; followed by 30 cycles of 94 °C for 1 min, 60 °C for 1 min, and 72 °C for 1 min; and finally an extension step executed at 72 °C for 7 min.

Serial dilution and plating methods were used to isolate the LAB isolates from various sources (Supplementary Table S1), and were discriminated using a MALDI Microflex LT mass spectrometer (Bruker Daltonics, Bremen, Germany), as previously described [44]. This was followed by species-specific PCR and *mutL* sequencing.

### 2.5. WGS of BCRC 06B0048 and Calculation of dDDH and Phylogenomic Tree Analysis

Genomic DNA was extracted using the EasyPrepHY genomic DNA extraction kit (Biotools Co. Ltd., Taipei, Taiwan) in accordance with the manufacturer’s protocols. The draft genomes of strain BCRC 06B0048 was sequenced from an Illumina paired-end library with an average insert size of 350 bp by using an Illumina HiSeq4000 platform with the PE 150 strategy at Beijing Novogene Bioinformatics Technology Co., Ltd. (Beijing, China). The resulting raw reads were assembled de novo using CLC Genomics Workbench version 20.0 (QIAGEN). The dDDH value and the phylogenomic tree were estimated and reconstructed using the TYGS server [45].

### 2.6. Differentiation of Strain BCRC 06B0048 and BCRC 11053^T^ Based on Biochemical and Chemotaxonomic Characteristics

Profiles of carbohydrate fermentation and enzymic activities were determined using the API 50 CH and API ZYM systems (bioMérieux, Marcy-l’Étoile, France), respectively, according to the manufacturer’s instructions. Whole-cell fatty acids were analyzed as fatty acid methyl esters (FAMEs) with the Sherlock Microbial Identification System (MIDI, Inc., Newark, NJ, USA), as described previously [46].

## 3. Results and Discussion

The gold standard marker for prokaryotic species identification has long been the 16S rRNA gene. Strains of a single species commonly exhibit 98.7% sequence identity with the 16S rRNA gene [47]. However, the 16S rRNA gene exhibits poor resolution in closely related species groups; for example, the *Lentilactobacillus buchneri*, *Lacticaseibacillus casei*, *L. plantarum*, and *Latilactobacillus sakei* groups [48,49,50,51,52]. Accordingly, researchers have employed comparative sequences of the housekeeping genes *dnaJ*, *dnaK*, *hsp60*, *pheS*, *recA*, *rpoA*, *rpoB*, and *tuf* to distinguish the members of the *L. plantarum* group [11,45,51,53,54]. Among them, *recA* exhibited the highest resolution at the interspecific level, along with relatively low sequence identities (75.6–93.0%; average: 80.6%; Supplementary Table S1). Moreover, researchers have established species-specific PCR primers that are targeted to the variable regions of the *recA* gene for species identification of *L. argentoratensis*, *L. paraplantarum*, *L. pentosus*, and *L. plantarum* [9,48]. However, because several phylogenetically closely related novel *L. plantarum* group species have been proposed in recent years [7,8], the usability and specificity of *recA* gene–based PCR and sequencing as well as species-specific primers should be reassessed through in silico methods. To identify highly distinguished taxonomic markers with deep-level phylogeny or species-specific genes according to the gain and loss of variable regions, the use of genome comparison methods have been applied to resolve the phylogenetically and phenotypically closely related species; this serves as an alternative to the conventional universal phylogenetic targets [55,56,57,58,59,60].

**Figure 1:**
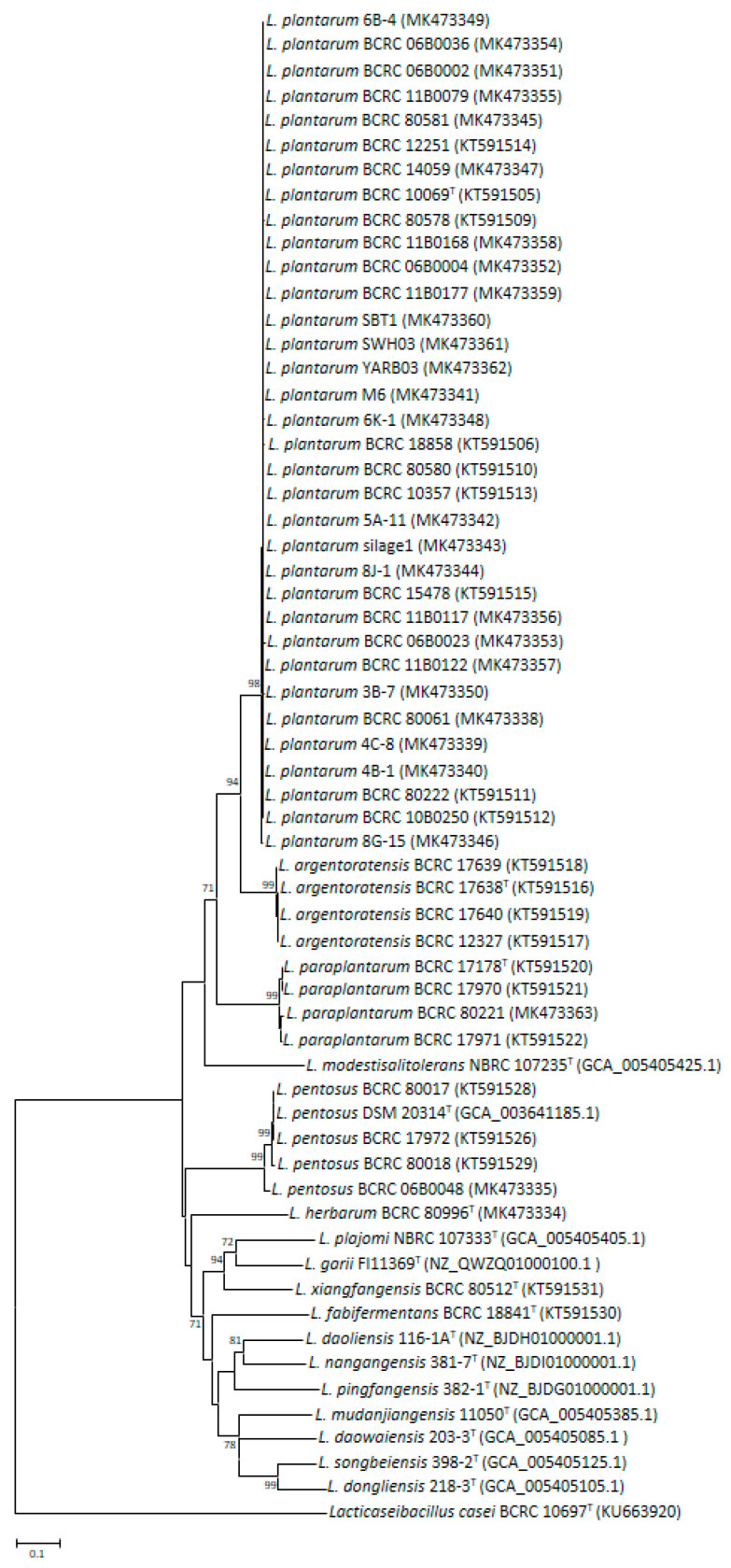
Phylogenetic tree of 57 *L. plantarum* group strains based on *mutL* sequences. The tree was constructed with the neighbour-joining method. Genetic distances were computed by Kimura’s two-parameter model. *L. casei* was included as an outgroup. Only bootstrap percentages above 70% are shown (based on 1000 replications). The scale bar represents 0.1% sequence divergence.

The genomic data of the *L. plantarum* group species exhibited sequences of high quality, as directly evidenced by the relatively small number of contigs (median, 48; Supplementary Table S2); we thus used the aforementioned data to execute our subsequent comparative genomic analyses. The pan genomic analysis of *L. plantarum* and its most closely related species was performed on the cano-wgMLST_BacCompare analytics platform; our derived results indicated that the pan genome contained 9382 genes, of which 13.12% (1231 genes) were present in all of the tested genomes. Subsequently, the 774 genes with the largest allele size were selected as the taxonomic markers, and target genes with high discrimination levels were developed by comparing their sequence identities, including maximum, minimum, and mean values within the *L. plantarum* group; the results revealed that *csbB, morA, murI, mutL, ntpJ, rutB, trmK, ydaF,* and *yhhX* genes were the most promising candidates (Supplementary Table S1). *mutL* encodes a DNA mismatch repair protein that has an essential role in the maintenance of genomic stability by correcting DNA replication error [61]. It is regarded as a crucial taxonomic marker for species- and strain-level differentiation in the *L. casei* group [44,62,63]. Thus, the discrimination power of *mutL* in the *L. plantarum* group merits further evaluation. A degenerate primer pair (LpmutL-F/R) was used to successfully amplify a PCR amplicon that comprised approximately of 1000 bp of *mutL* from *L. plantarum* group strains; this product was subsequently employed for sequencing. Among the type strains, the average sequence identity of *mutL* was 66.6% (61.6–85.6%), which was clearly superior to that of the 16S rRNA gene (96.7–100%; average: 98.4%) (Table 2) and conventional phylogenetic markers (Supplementary Table S1) and could be applied to effectively separate tested strains into various species clusters with a high bootstrap value in the phylogenetic tree (Figure 1). Consequently, species-specific primers for four species in the *L. plantarum* group were designed (Table 3); these primers could successfully generate a single specific amplicon (319, 115, 176, and 385 bp) when they were used in PCR processes with DNA from reference strains of *L. argentoratensis*, *L. paraplantarum*, *L. plantarum*, and *L. pentosus* (Table 1), with no cross-reaction against nontarget LAB species. Furthermore, 20 LAB isolates were isolated from various sources (animal feces, fermented foods, and silage), and MALDI-TOF MS indicated that 13 of them were related to *L. plantarum*; these LAB isolates were next identified to the species-level of *L. plantarum* by using *mutL*-based species-specific PCR and sequencing (Supplementary Table S3). On the other hand, all 33 *L. plantarum* strains could be assigned to a combination of 16 different haplotypes using *mutL* gene sequence, which was comparable with that of other conventional MLST targets to distinguishing between strains in *L. plantarum* species [64,65].

**Figure 2:**
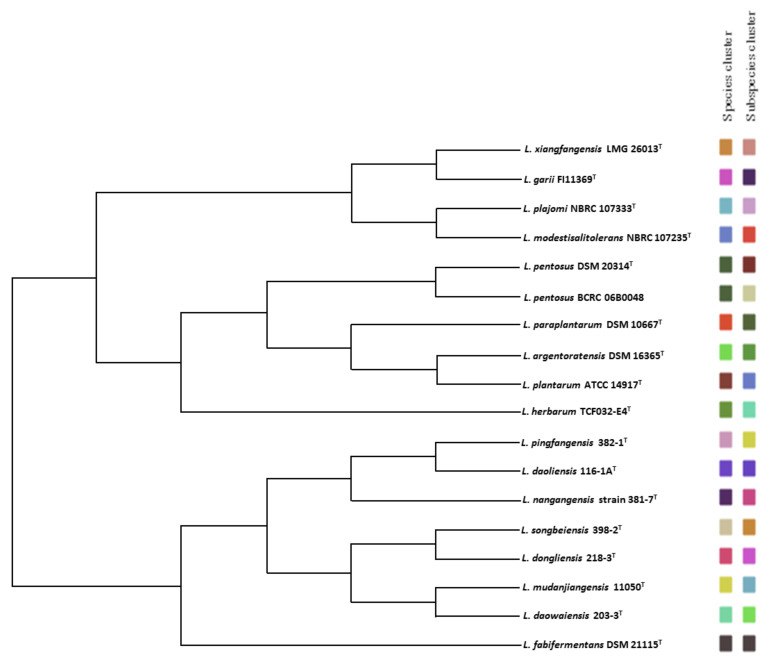
Phylogenomic tree of the 18 *L. plantarum* group strains available on the TYGS database. The tree was inferred with FastME 2.1.6.1 from GBDP distances calculated from genome sequences. The branch lengths are scaled in terms of GBDP distance formula d5. The tree was rooted at the midpoint. Accession numbers of the genome sequences used for the reconstruction are shown in Supplementary Table S2.

We note that the BCRC 06B0048 strain, which was originally identified as *L. pentosus*, had relatively low *mutL* sequence identities (97.0%) with the type strain DSM 20314^T^; moreover, the *mutL*-based phylogenetic tree indicates that the five strains of the species *L. pentosus* could be divided into two subclusters and that the nodes exhibited high bootstrap values (99%; Figure 1). Traditionally, subspecies-level differentiation mainly relied on the phenotypic and genotypic characteristics. Nevertheless, genome-based taxonomic designation for subspecies with the use of 79% dDDH has been suggested [66]. This approach has been successfully applied to propose and officially validate novel bacterial subspecies such as *Bifidobacterium catenulatum*, *B.* *gallinarum B. thermacidophilum*, and *L. reuteri* [67,68]. The draft genome size of the BCRC 06B0048 strain was 3.7 Mb (G+C content: 46.2 mol%), and it contained 3,293 protein-coding genes (Supplementary Table S2). The dDDH value between the BCRC 06B0048 and DSM 20314^T^ strains was 78.1%, which is below the subspecies delineation threshold value. The phylogenomic tree revealed that the 17 type strains of the *L. plantarum* group were clearly differentiated into distinct species clusters, and the species *L. pentosus* could also be divided into two independent subclusters (Figure 2). In addition, strain BCRC 06B0048 has also shown several differential characteristics from the *L. pentosus* type strain BCRC 11053^T^ (Supplementary Table S4). These results indicate that strain BCRC 06B0048 has the potential for reclassification as an independent subspecies of *L. pentosus.*

## 4. Conclusions

In this study, we successfully applied a genome-based analysis method to assist in the selection of the promising target genes for a closely related species group by employing the cano-wgMLST_BacCompare analytics platform. The results suggest that the genome-wide taxonomic marker of *mutL* is an excellent phylogenetic target for precisely discriminating and identifying *L. plantarum* and related taxa through the use of direct sequencing as well as species-specific PCR assays. According to the genotypic and phenotypic characteristics, we deduced that strain BCRC 06B0048 may represent an undescribed subspecies of *L. pentosus*. We will endeavor to integrate polyphasic and combined with the genomic strategies to describe novel subspecies in the future.

## Figures and Tables

**Table 1:** Strains used in this study and specificity tests with *mutL*-targeting PCR assays.

No.	Species	Strain No.	Other Destination	Species-Specific PCR Assays			
				spLplan-F/R	spLarg-F/R	spLpara-F/R	spLpen-F/R
1	*L. plantarum*	BCRC10069^T^	ATCC 14917^T^	+	−	−	−
2		BCRC 10357	ATCC 8014	+	−	−	−
3		BCRC 12251	ATCC 10241	+	−	−	−
4		BCRC 14059	ATCC 10012	+	−	−	−
5		BCRC 15478	NCDO 1193	+	−	−	−
6		BCRC 18858	NRIC 1943	+	−	−	−
7		BCRC 80061	−	+	−	−	−
8		BCRC 80222	CECT 5787	+	−	−	−
9		BCRC 80578	NCDO 772	+	−	−	−
10		BCRC 80580	CICC 6026	+	−	−	−
11		BCRC 80581	CICC 20764	+	−	−	−
12		BCRC 06B0002	−	+	−	−	−
13		BCRC 06B0006	−	+	−	−	−
14		BCRC 06B0023	−	+	−	−	−
15		BCRC 06B0036	−	+	−	−	−
16		BCRC 11B0079	−	+	−	−	−
17		BCRC 11B0117	−	+	−	−	−
18		BCRC 11B0122	−	+	−	−	−
19		BCRC 11B0168	−	+	−	−	−
20		BCRC 11B0177	−	+	−	−	−
21	*L. argentoratensis*	BCRC17638^T^	DSM 16365^T^	−	+	−	−
22		BCRC 17639	CCUG 50788	−	+	−	−
23		BCRC 17640	CCUG 50789	−	+	−	−
24	*L. paraplantarum*	BCRC 17178^T^	DSM 10667^T^	−	−	+	−
25		BCRC 17970	ATCC 10776	−	−	+	−
26		BCRC 17971	ATCC 700210	−	−	+	−
27		BCRC 80221	CECT 5783	−	−	+	−
28	*L. pentosus*	BCRC 11053^T^	ATCC 8041^T^	−	−	−	+
29		BCRC 17972	LMG 9210	−	−	−	+
30		BCRC 80017	JCM 8334	−	−	−	+
31		BCRC 80018	JCM 8335	−	−	−	+
32		BCRC 06B0048	−	−	−	−	+
33	*L. daoliensis*	116-1A^T^	NCIMB 15181	−	−	−	−
34	*L. pingfangensis*	382-1^T^	NCIMB 15187	−	−	−	−
35	*L. daowaiensis*	203-3^T^	NCIMB 15183	−	−	−	−
36	*L. nangangensis*	381-7^T^	NCIMB 15186	−	−	−	−
37	*L. herbarum*	BCRC 80996^T^	DSM 100358^T^	−	−	−	−
38	*L. fabifermentans*	BCRC 18841^T^	LMG 24284^T^	−	−	−	−
39	*L. plajomi*	BCRC 80928^T^	NBRC 107233^T^	−	−	−	−
40	*L. xiangfangensis*	BCRC 80512^T^	LMG 26013^T^	−	−	−	−
41	*L. modestisalitolerans*	BCRC 80927^T^	NBRC 107235^T^	−	−	−	−
42	*L. dongliensis*	218-3^T^	NCIMB 15184^T^	−	−	−	−
43	*L. songbeiensis*	398-2^T^	NCIMB 15189^T^	−	−	−	−
44	*L. mudanjiangensis*	11050^T^	LMG 27194^T^	−	−	−	−
45	*L. acidophilus*	BCRC 10695^T^	ATCC 4356^T^	−	−	−	−
46	*L. casei*	BCRC 10697^T^	ATCC 393^T^	−	−	−	−
47	*L. crispatus*	BCRC 14618^T^	ATCC 33820^T^	−	−	−	−
48	*L. curvatus*	BCRC 12189^T^	DSM 20019^T^	−	−	−	−
49	*L. delbrueckii* subsp. *bulgaricus*	BCRC 10696^T^	ATCC 11842^T^	−	−	−	−
50	*L. gallinarum*	BCRC 17266^T^	ATCC 33199^T^	−	−	−	−
51	*L. gasseri*	BCRC 14619^T^	ATCC 33323^T^	−	−	−	−
52	*L. paracasei*	BCRC 12248^T^	ATCC 25302^T^	−	−	−	−
53	*L. rhamnosus*	BCRC 10940^T^	ATCC 7469^T^	−	−	−	−
54	*L. sakei*	BCRC 14622^T^	ATCC 15521^T^	−	−	−	−
55	*L. taiwanensis*	BCRC 17755^T^	JCM 18086^T^	−	−	−	−
56	*L. ultunensis*	BCRC 17714^T^	DSM 16047^T^	−	−	−	−

BCRC, Bioresource Collection and Research Center at Food Industry Research and Development Institute, Taiwan; +, PCR products with specific primer detected; −, PCR products with specific primer not detected.

**Table 2:** Identities of *mutL* and 16S rDNA sequences between the type strains of the *L. plantarum* group.

No.	Species	1	2	3	4	5	6	7	8	9	10	11	12	13	14	15	16	17
1	*L. plantarum* BCRC 10069^T^		85.6	76.1	69.4	64.9	64.0	64.5	67.0	64.8	67.9	64.6	65.9	68.1	66.8	65.0	65.7	64.9
2	*L. argentoratensis* BCRC 17638^T^	100		73.6	68.6	65.1	64.4	64.6	66.2	65.4	66.6	64.7	65.6	68.0	66.4	63.8	65.1	64.4
3	*L. paraplantarum* BCRC 17178^T^	99.7	99.7		69.3	66.7	66.2	66.5	66.4	66.5	68.1	66.2	65.4	68.8	65.2	67.0	66.1	66.6
4	*L. pentosus* BCRC 11053^T^	99.9	99.9	99.8		65.4	65.9	66.5	66.5	63.3	66.4	64.1	65.8	67.9	64.5	65.8	67.5	66.1
5	*L. daoliensis* 116-1A^T^	99.0	99.0	99.0	99.1		71.6	65.6	65.9	73.8	63.7	66.4	64.5	64.3	65.1	66.0	66.3	65.9
6	*L. pingfangensis* 382-1^T^	99.0	99.0	99.2	99.1	99.7		70.4	64.5	73.0	66.3	65.9	65.5	66.5	66.2	65.8	66.9	69.9
7	*L. daowaiensis* 203-3^T^	98.9	98.9	98.8	98.9	98.5	98.5		66.0	66.1	64.3	68.2	62.7	66.1	67.0	70.2	69.5	71.1
8	*L. garii* FI11369^T^	98.9	98.9	98.9	98.9	98.5	98.5	98.7		61.6	64.9	65.4	69.6	73.3	64.6	66.1	66.3	64.3
9	*L. nangangensis* 381-7^T^	98.9	98.9	99.0	99.0	99.9	99.8	98.6	98.6		63.8	66.7	63.4	65.4	64.3	66.0	67.6	67.6
10	*L. herbarum* BCRC 80996^T^	98.9	98.9	98.9	98.9	98.3	98.5	98.0	98.0	98.4		64.0	64.9	67.7	62.4	64.0	64.6	64.3
11	*L. fabifermentans* BCRC 18841^T^	98.9	98.9	98.9	98.9	98.5	98.6	98.5	98.5	98.5	98.3		62.7	64.5	65.0	66.3	67.3	66.1
12	*L. plajomi* BCRC 80928^T^	98.8	98.8	98.5	98.7	98.0	97.8	98.2	99.0	97.9	97.7	98.1		68.1	64.3	65.4	64.8	62.8
13	*L. xiangfangensis* BCRC 80512^T^	98.7	98.7	98.7	98.8	99.0	99.0	98.4	99.4	99.1	98.0	98.5	98.7		65.6	68.0	70.5	68.6
14	*L. modestisalitolerans* BCRC 80927^T^	98.5	98.5	98.4	98.5	97.8	97.7	97.8	98.7	97.8	97.5	97.5	98.9	98.4		67.0	67.7	66.0
15	*L. dongliensis* 218-3^T^	98.0	98.0	97.8	97.9	97.9	98.1	98.3	97.6	98.0	97.3	97.5	96.9	97.9	96.8		80.2	69.9
16	*L. songbeiensis* 398-2^T^	97.9	97.9	97.9	98.0	98.1	98.2	98.2	97.5	98.2	97.4	97.6	96.9	98.0	96.7	99.8		70.3
17	*L. mudanjiangensis* 11050^T^	97.8	97.8	97.9	97.9	97.9	98.1	98.5	97.6	98.0	97.6	97.5	96.9	97.5	96.8	98.7	98.8	

The values on the upper right are the similarities between *mutL* sequences, and the values on the lower left are the similarities between 16S rRNA sequences.

**Table 3:** Species-specific primers used in this study.

No.	Primer	Target	Sequence (5′–3′)	Amplicon Size (bp)
1	spLarg-F	*L. argentoratensis*	CCTTTGGTGAACCCGCTGAA	319
	spLarg-R		AGTTCGGCTAATAGTGGCAA	
2	spLpara-F	*L. paraplantarum*	TCAGGTGGCGGATAAGACTAC	115
	spLpara-R		GGTTGCCGAMGTGGCGTCA	
3	spLpen-F	*L. pentosus*	CCTCCGCTGAACCAATCATG	385
	spLpen-R		TTCAGGACATCACTGGTGGG	
4	spLplan-F	*L. plantarum*	GCGRTTGTTCCGTCAGAAT	176
	spLplan-R		CTTGCAGCCGTGCTGGTTT	

## Data Availability

The sequencing data has been uploaded to GenBank (KT591505~KT591231, MK473334~MK473363, and JAGHKR000000000).

## Supplementary Materials

The following are available online at https://www.mdpi.com/article/10.3390/microorganisms9081570/s1, Table S1: Interspecific analysis of the genome-based selection targets within the *L. plantarum* group. Table S2: Genomic characteristics of *L. plantarum* group type strains and BCRC 06B0048. Table S3: Identification of *L. plantarum* isolates from various sources using MALDI-TOF MS, species-specific PCR and *mutL* sequencing methods. Table S4: Differential characteristics between the *L. pentosus* BCRC 06B0048 and BCRC 11053^T^.
